# Antimicrobial Prescriptions for Dogs in the Capital of Spain

**DOI:** 10.3389/fvets.2018.00309

**Published:** 2018-12-04

**Authors:** Bárbara Gómez-Poveda, Miguel A. Moreno

**Affiliations:** ^1^Department of Animal Health, Faculty of Veterinary, Complutense University, Madrid, Spain; ^2^VISAVET Center, Complutense University, Madrid, Spain

**Keywords:** antibiotics, pets, survey, beta lactams, prescriptions, conditions

## Abstract

**Objective:** To characterize antimicrobial prescription patterns for dogs in veterinary practices in Spain using the city of Madrid as a model.

**Design:** Retrospective survey.

**Settings:** Dogs attending veterinary practices in the city of Madrid in 2017 were enrolled.

**Subjects:** Three hundred dogs from 30 veterinary practices randomly selected from a set of 388 practices grouped by zip code. The inclusion criterion for dogs was treatment with antibiotics within a few days of the data collection day.

**Results:** For the 300 dogs enrolled, 374 treatments with antimicrobials were recorded, 62.8% (235/374) were veterinary medicinal products and 37.2% (139/374) human medicinal products. The main route of administration was oral (209/374; 55.9%) followed by parenteral (100/374; 26.7%) and topical (65/374; 17.4%). Sixty-five dogs (21.7%) received a perioperative antimicrobial treatment, mainly associated with female obstetrical surgery (19/65; 29%), while 78.3% (235/300) received a pharmaceutical treatment mainly for skin (72/235; 30.6%), respiratory (47/235; 20%), or digestive (41/235; 17.4%) diseases. The most frequently used antimicrobials were beta-lactams for oral (119/209) and parenteral (79/100) administration, especially the combination amoxicillin with clavulanic acid (83/209; oral), amoxicillin alone (42/100; parenteral), and aminoglycosides (32/65) for topical use. Diagnostic confirmation with culture was carried out on only 13 out of 235 dogs receiving therapeutic treatment and nine underwent an antimicrobial susceptibility test. In addition, cytology was performed in 15 dogs.

**Conclusions:** The pattern of antimicrobial prescriptions for dogs in our study was quite similar to that previously described in several European countries, and encompassed the same two highly interconnected key features: major use of amoxicillin with clavulanic acid and a very low level of antimicrobial susceptibility testing before prescription. Consequently, we recommend that the measures for rationalizing antimicrobial prescription for dogs in Spain should follow those implemented in other countries, especially confirming the diagnosis and promoting the use of hygiene measures by owners.

## Introduction

Antimicrobial resistance is currently one of the leading public health risks and antimicrobial usage one of its key drivers, both in humans and animals.

Antimicrobial resistance in animal bacteria (zoonotic, pathogenic for animals or commensal) is of great concern, especially if resistant bacteria can be spread to humans. Foodborne transmission is the most frequently studied route, but some authors have raised awareness about the increasing importance of direct contact transmission with pets (1, 2) (for veterinary surgeons and owners, especially children) and food animals (for workers, veterinary surgeons, etc.).

Antimicrobials (AM) are frequently prescribed for companion animals in the treatment of various conditions. Due to high public health concern, there are an increasing number of guidelines for prudent or responsible use of antimicrobials (see for example World Health Organization (http://www.who.int/foodsafety/publications/cia_guidelines/en/); Federation of Veterinarians of Europe (https://www.fecava.org/sites/default/files/files/fve_antimicrobials_pets_final_small.pdf) or Responsible Use of Medicines in Agriculture Alliance (https://www.ruma.org.uk/antimicrobials/guidelines/). Although factors influencing antibiotic prescribing habits of veterinary surgeons are not universal (3), those for veterinary surgeons of companion and food animals are quite similar. They include self-training, literature reviews, official reports, and commercial information (3).

Some human medicinal products (MP) containing antimicrobials are also used for companion animals (2), according to the prescribing “cascade” procedure (4) (articles 10 and 11 of Directive 2001/82/EC). Uses deviating from the Summary of Product Characteristics (SPC) are called off-label use (5). Current regulation of veterinary medicinal products (VMP) in the EU allows veterinary surgeons (under certain circumstances, to avoid causing unacceptable suffering to diseased animals, and under their own responsibility) to prescribe human MP for animals. A reflection paper on off-label use has been publishe by the European Medicines Agency (EMA) (5).

In the European Union (EU), sales of VMP containing antimicrobials have been compiled by the EMA from data provided by national authorities since 2010. This was in response to a 2008 mandate from the EU Commission (6). The EMA publish a yearly European Surveillance of Veterinary Antimicrobial Consumption (ESVAC) report on antimicrobial sales that mostly covers food animals and produces a national indicator relating antimicrobial sales and animal biomass expressed as milligram of antimicrobials per population correction unit. This is an overall indicator covering the major food animal species but is not specific for any species. Although the authorized data sheets for dog products, typically in the form of tablets but also injectable, are provided by some participating countries, the Statistical Office of the European Union (Eurostat) does not have accurate data on dog and cat populations, and consequently, they are not included in the national indicator mentioned above. According to the last ESVAC report (6), sales of tablets accounted for <8% of total antimicrobial sales in all the countries, except Iceland, Finland, Norway, and Sweden.

Total amounts of antimicrobials (sales or consumption data) are not the only approach for understanding the selective pressure for antimicrobial resistant bacteria. Complementary information such as patterns of use of antimicrobials according to animal species, conditions, etc. is also of value. Some information about these patterns in dogs already exists, especially from the UK (7–9), but also from Finland (10), Italy (11), and Australia (12). There are no current data available for Spain although, according to the 2016 ESVAC report (6), Spain was ranked as the second EU country by antimicrobial sales in animals.

The aim of this survey was to characterize antimicrobial prescriptions in dogs in a random sample of veterinary practices in the city of Madrid, Spain, assuming that they could be used as a rationale estimate of prescriptions throughout the country.

## Material and Methods

### Sampling Frame

A sampling frame of 388 veterinary practices treating dogs in the city of Madrid (comprising about 1,000 practitioners) was constituted in December 2016 with data from the websites of the Official Veterinary Professional Association of Madrid (https://www.colvema.org/sac_lis_clinicas.asp) and a phone book web page (www.paginasamarillas.es). Veterinary practices were grouped by zip code, obtaining 52 zip codes that had at least one veterinary practice (from 1 to 14 veterinary practices per zip code).

### Sampling Design

From these 52 zip codes, 30 were randomly selected and then one veterinary practice was also randomly selected from each zip code. Finally, 10 dogs attending the 30 veterinary practices who agreed to collaborate with the survey were included in the study on the basis of having recently received an antimicrobial prescription prior to being contacted during 2017.

### Data Collection

Veterinary surgeons in charge of the enrolled veterinary practices were contacted by phone to confirm their willingness to participate in the survey. After verbal agreement, a physical meeting at their facilities was convened for compiling information from their records of case histories.

### Data Collection Form

The data collection form (in Spanish and available as Supplementary Material) contained questions regarding the dog (sex, breed, birth date, and weight), current condition (date, clinical signs, diagnostic, bacteriological culture, antimicrobial susceptibility test, and other diagnostic tests) and the antimicrobial prescriptions or administration in the practice (commercial name, active substance, administration route, posology, pharmaceutical form, and prescription type), considering all antimicrobials prescribed on the same record. Amoxicillin plus clavulanic acid was considered a single drug for the purposes of data collection. Any personal information regarding the pets' owners was also recorded.

### Data Recording and Analysis

Data were recorded in Microsoft Excel and a descriptive analysis performed with the same program and with the IBM SPSS Statistics software, version 22.

### Conditions (Treatment Indication)

We distinguished two main uses of MP. When a dog received a MP for a condition, this was classified as a therapeutic treatment. Whereas when a dog received the MP as part of a surgical procedure (administered prior, during or after the surgical procedure), the administration was classified as prophylactic. In addition, we grouped therapeutic treatments according to the main systems/organs involved (skin, mouth, digestive tract, respiratory tract, ear, eye, urinary, and other), and prophylactic treatments according to similar medical criteria (obstetrics, male genitourinary operation, odontology, traumatology, dermatology, and other).

### Antimicrobial Prescription Assessment

The recorded use of all VMP was checked against their respective Summary of Product Characteristics (https://cimavet.aemps.es/cimavet/medicamentos.do) for compliance with target species, indications for use (condition), and posology (dosage and duration).

## Results

### Sample Description

Overall during the study, we contacted 50 veterinary practices to recruit the 30 practices who eventually agreed to participate in this survey. The 30 practices included in the study belonged to 29 of the 30 zip codes randomly selected in the original sampling. Because none of the veterinary practices belonging to one zip code was able to participate, this zip code was replaced by another on the list. In summary, 18 of the 30 veterinary practices originally selected for the study were willing to participate, whereas 12 were replaced.

Of the 300 dogs participating in the survey, 174 (58%) were male and 42% (126/300) female. Their age ranged from three months to 17 years (mean = 5.9 years; standard deviation = 4.5). The dogs were classified into 49 breeds, with 93 of the dogs (30.7%) being crossbreeds.

### Diagnostic Tests for Bacterial Infection

Bacteriological culture had been performed for 5.5% (13/235) of the dogs receiving a therapeutic treatment (six ear, three urinary, two skin, and two digestive conditions) and an antimicrobial susceptibility test for 3.8% of the dogs (9/235) (five ear, three urinary, and one skin conditions). Cytology testing was performed in 6.4% (15/235) cases (five ear, four skin, one urinary, and five miscellaneous conditions).

### Medicinal Products

We documented 374 MP containing antimicrobials from the medical records of 300 dogs (Table 1), prescribed between January and July 2017. Two hundred and thirty-two dogs received one product, 63 dogs received two products, four dogs received three products and one dog four products.

**Table 1:** Distribution of antimicrobials of 374 medicinal products (MP) prescribed to 300 dogs (Madrid City) according to authorization for Veterinary (VMP) or Human (HMP) use.

**Antimicrobials**	**VMP**	**HMP**	**Total MP**	**% over 374 MP**	**Dogs**	**% over 300 dogs**
Beta-lactams	**146**	**55**	**201**	**53.7**	**187**	**62.3**
Amoxicillin-clavulanic acid	61	42	103	27.5	91	30.3
Amoxicillin/ampicillin	43	1	44	11.8	44	14.7
Benzylpenicillin^**^	2		2	0.5	2	0.7
Cefalexin	29	12	41	11.0	39	13.0
Cefovecin	10		10	2.7	10	3.3
Cefquinome	1		1	0.3	1	0.3
Fluoroquinolones	**41**	**5**	**46**	**12.3**	**44**	**14.7**
Marbofloxacin	23		23	6.1	23	7.7
Enrofloxacin	18	1	19	5.1	17	5.7
Ciprofloxacin		4	4	1.1	4	1.3
Aminoglycosides	**8**	**33**	**41**	**11.0**	**41**	**13.7**
Neomycin	6	13	19	5.1	19	6.3
Tobramycin		12	12	3.2	12	4.0
Gentamicin		5	5	1.3	5	1.7
Dihydrostreptomycin	2	3	5	1.3	5	1.7
Imidazole derivatives	**8**	**28**	**36**	**9.6**	**36**	**12.0**
Metronidazole	8	28	36	9.6	36	12.0
Polymyxins	**12**	**3**	**15**	**4.0**	**15**	**5.0**
Polymyxin B	12	3	15	4.0	15	5.0
Tetracyclines	**8**	**6**	**14**	**3.7**	**14**	**4.7**
Doxycycline	8	6	14	3.7	14	4.7
Macrolides and lincosamides	**7**	**8**	**15**	**4.0**	**15**	**5.0**
Spiramycin^***^	6	3	9	2.4	9	3.0
Clindamycin		3	3	0.8	3	1.0
Azithromycin		2	2	0.5	2	0.7
Tylosin	1		1	0.3	1	0.3
Sulphonamides	**3**	**8**	**11**	**2.9**	**11**	**3.7**
Sulfadoxine -trimethoprim	3	5	8	2.1	8	2.7
Formosulfathiazol		3	3	0.8	3	1.0
Others	**10**	**2**	**12**	**3.2**	**12**	**4.0**
Florfenicol	6		6	1.6	6	2.0
Fusidic acid	4		4	1.1	4	1.3
Mupirocin		2	2	0.5	2	0.7

*Combinations: Metronidazole-spiramycin; sulfadoxine-trimethoprim; Benzylpenicillin-dihydrostreptomycin; polymyxin B-neomycin; formosulfathiazol-dihydrostreptomycin-neomycin*;

** *Always in combination with streptomycin*;

*** *Always in combination with metronidazole*.

Based on the data sheets, 62.8% (235/374) of the products were for veterinary use and 37.2% (139/374) were for human use (including 15 extemporaneously prepared products; Table 1).

The most common administration route was oral (209/374; 55.9%; Table 2), followed by parenteral (100/ 374; 26.7%; Table 3), and topical (65/374; 17.4%; Table 4).

**Table 2:** Distribution of antimicrobials of 209 oral medicinal products according to organ/system (conditions) treated, on 300 urban dogs (Madrid City).

	**Respiratory**	**Urinary**	**Skin**			**Eye**	**Ear**	**Digestive**		**Surgical**	**Others**
**Antimicrobial class**			**Dermatitis**	**Bite**	**Folliculitis**	**Corneal Ulcers**	**Otitis**	**Gastroenteritis**	**Gingivitis**		
Single (194)	35	14	21	9	3	2	10	25	5	31	39
Combinations (15)		1		1				5	2	3	3
Beta-lactams	**20**	**7**	**17**	**6**	**3**		**5**	**2**	**5**	**20**	**28**
Amoxicillin-clavulanic acid	20	7	5	3			3	2	5	20	17
Amoxicillin/ampicillin			1								1
Cephalexin			11	3	3		2			7	10
Fluoroquinolones	**4**	**6**	**3**	**2**		**1**	**5**	**1**		**1**	**7**
Marbofloxacin	1		3	2		1	3				6
Enrofloxacin	3	4					1	1			1
Ciprofloxacin		2					1			1	
Nitroimidazoles				**1**				**22**			**1**
Metronidazole				1				22		1	1
Tetracyclines	**9**	**1**				**1**				**1**	**2**
Doxycycline	9	1				1				1	2
Macrolides and lincosamides	**2**		**1**							**1**	**1**
Clindamycin			1							1	1
Azithromycin	2										
Combinations											
Sulfadoxine-trimethoprim		1						1			1
Metronidazole-spiramycin				1				1	2	3	2
Formosulfathiazol-dihydrostreptomycin-neomycin								3			

**Table 3:** Distribution of antimicrobials of 100 parenteral medicinal products according to organ/system (conditions) treated, on 300 urban dogs (Madrid City).

	**Respiratory**	**Urinary**	**Skin**		**Ear**	**Digestive**	**Surgical**	**Others**
**Antimicrobial class**			**Dermatitis**	**Bite**	**External Otitis**	**Gastroenteritis**		
Single (93)	19	4	6	4	1	11	31	17
Combinations (7)	1					6		
Beta-lactams	**17**	**3**	**5**	**4**	**1**	**6**	**29**	**14**
Amoxicillin-clavulanic acid	7			1		3	9	1
Amoxicillin/ampicillin	6	2	2	2	1	3	17	9
Cephalexin	3			1				1
Cefovecin	1	1	3				2	3
Cefquinome							1	
Fluoroquinolones	**2**	**1**	**1**			**2**	**2**	**3**
Marbofloxacin	1						1	
Enrofloxacin	1	1	1			2	1	3
Ciprofloxacin								
Nitroimidazoles						**2**		
Metronidazole						2		
Macrolides and lincosamides						**1**		
Tylosin						1		
Combinations								
Benzylpenicillin- dihydrostreptomycin	1					1		
Sulfadoxine-trimethoprim						5		

**Table 4:** Distribution of antimicrobials of 65 topical medicinal products according to administration route and conditions treated, on 300 urban dogs (Madrid City).

	**Ocular**			**Otic**	**Cutaneous**		
**Antimicrobial class**	**Conjunctivitis**	**Corneal ulcers**	**Others**	**Otitis**	**Dermatitis**	**Ulcers**	**Others**
Single (63)	13	5	4	24	9	3	5
Combinations (2)		1		1			
Fluoroquinolones				**5**			
Marbofloxacin				5			
Aminoglycosides	**13**	**5**	**2**		**6**	**2**	**5**
Neomycin	2	1	1		5	2	5
Tobramycin	7	4	1				
Gentamicin	4				1		
Polymixyns			**2**	**11**			
Polymyxin B			2	11			
Others							
Florfenicol				6			
Fusidic acid				2	2		
Mupirocin					1	1	
Combinations							
Polymyxin B-neomycin		1		1			

### Antimicrobials

Of the 374 products, 93.6% (350/374) contained one single antimicrobial, while the remaining 6.4% (24/374) combined two (metronidazole - spiramycin; sulfadoxine - trimethoprim; benzylpenicillin - dihydrostreptomycin and polymyxin B - neomycin) or three (formosulfathiazol—dihydrostreptomycin - neomycin).

The 374 products contained 26 different antimicrobials (Table 1), with beta-lactams (201/374; 53.7%) being the most widely used antibiotic class by far, followed by fluoroquinolones (46/374; 12.3%), aminoglycosides (41/374; 11%), and imidazole derivatives (36/374; 9.6%). Of the active ingredients, amoxicillin with clavulanic acid was the most common, followed by amoxicillin, cephalexin, and metronidazole. Four of the identified antimicrobials (ciprofloxacin, tobramycin, azithromycin, and mupirocin) were not authorized for veterinary use in Spain.

The distribution of antimicrobial treatments according to the administration route showed that most of them fell into systemic (oral or parenteral) or topical (skin, eye, ear) use. Beta-lactams, macrolides, lincosamides, tetracyclines, sulphonamides, trimethoprim, and metronidazole were always used systemically (Tables 2, 3), whereas polymyxins, phenicols, fusidic acid, and mupirocin were only used topically (Table 4). Fluoroquinolones were mainly for systemic use but some topical products contained marbofloxacin. Aminoglycosides were mostly used topically, although streptomycin and neomycin were sporadically employed systemically.

### Conditions (Treatment Indication)

Two hundred and thirty-five out of 300 dogs (78.3%) received a therapeutic treatment with an antimicrobial product, whereas 65 out of 300 dogs (21.7%) received a prophylactic (perioperative) treatment (35 after surgery, 21 during the intervention and nine prior to surgery). Surgical procedures included the following interventions: obstetrical (19 of 65 dogs; 29%), male genitourinary (14/65; 22%), dental (8/65; 12%), skin (8/65; 12%), traumatological (6/65; 9%), and other (10/65; 15%; Table 5).

**Table 5:** Distribution of antimicrobials of 65 medicinal products for perioperative use according to type of surgery.

**Surgery**	**Amoxicillin with clavulanic acid**	**Amoxicillin**	**Cephalosporins**	**Fluoroquinolones**	**Others**
Obstetrics (19)	11	4	2 (cephalexin) 1 (cefovecin)		1 (metronidazole)
Genito-urinary male (14)	7	5	2 (cephalexin)		
Dental (8)	1	2	1 (cephalexin) 1 (cefovecin)		2 (metronidazole-spiramycin) 1 (clindamycin)
Skin (8)	4	2		1 (enrofloxacin)	1 (doxycycline)
Traumatology (6)	2	1	2 (cephalexin)	1 (marbofloxacin)	
Others (10)	4	3	1 (cefquinome)	1 (ciprofloxacin)	1 (metronidazole-spiramycin)

The most common general conditions for therapeutic use of antimicrobials were skin disorders (72/235; 30.6%), respiratory disorders (47/235; 20%), and digestive disorders (41/235; 17.4%). The specific diseases that were more frequent in skin were dermatitis (20/235; 8.5%) and pyoderma (9/235;3.8%); in the respiratory tract, kennel cough (39/235; 16.6%); in the digestive system, enteritis (20/235; 8.5%) and gastroenteritis (14/235; 6%); in the ear, external otitis (30/235; 12.8%); in the eye, conjunctivitis (12/235; 5.1%), and in the urinary tract, cystitis (15/235; 6.4%).

### Assessment of Prescription Compliance With the Summary of Product Characteristics (SPC)

The data recorded from the practitioners when using the 235 VMP were checked against the SPC for compliance with target species, indications for use (condition) and posology (dosage and duration; Table 6). Only 15 VMP did not list dogs as the target species (all of these were authorized for several other food animals). The condition treated was listed in the indication for use in 64.3% of the products, with the lowest compliance recorded for digestive disorders. Compliance with recommended dosage fluctuated between 42 and 94%, with overdosage (23%) more common than underdosage (12.8%). In summary, 40.4% of the VMP were used in accordance with the SPC. The use in dogs of human products was not evaluated.

**Table 6:** Assessment of compliance with the summary of the product characteristics (SPC) of 235 veterinary medicinal products (VMP) prescribed to 300 dogs.

**Condition**	**Target species**	**Condition**	**Dosage**	**Duration**	**All**	**Over dosage**	**Under dosage**
Skin (61)	98%	80%	70%	80%	49%	23%	7%
Digestive (24)	71%	29%	42%	71%	13%	25%	13%
Respiratory (40)	95%	60%	53%	78%	35%	18%	28%
Ear (31)	100%	90%	94%	97%	81%	3%	3%
Eye (2)	2/2^*^	0/2	1/2	2/2	0/2	0/2	1/2
Urinary (10)	90%	70%	50%	100%	40%	30%	20%
Others (17)	94%	65%	71%	82%	41%	18%	12%
Surgical (50)	94%	50%	48%	68%	24%	38%	12%
All (235)	**93.6%**	**64.3%**	**61.7%**	**79.6%**	**40.4%**	**23.0%**	**12.8%**

* *Figures having a denominator lower than 10 are not expressed as percentages*.

## Discussion

### Survey Design and Potential Biases

Veterinary teaching hospital records (10, 11) and veterinary practice electronic records in private databases (7–9) were used as sources of data in previous studies, none of which could be considered as census studies at their respective national level. Nevertheless, all of them were able to draw reasonable pictures of antimicrobial prescription in dogs that could be generalized to their countries. This survey was based on a random selection of 30 veterinary practices located in the city of Madrid but relied on the willingness of the practitioners to participate. This could have biased the sample in favor of those more likely to collaborate with the Veterinary Faculty or those specifically interested in the topic. Nonetheless, 60% (18/30) of the effective participants belonged to the random sample selected, whereas all but one of the remainder came from randomly selected zip codes. This gives some confidence that the sample was representative.

Most of the treatments were prescribed in winter and spring (from January to April 2017), which could have produced a seasonal bias in favor of the conditions that are more common during this period.

Finally, the information recorded on the case history of the dogs was quite diverse and many difficulties arose when we tried to cluster treatments based on clear indications, as mentioned in the materials and methods section. This might produce discrepancies when antimicrobials per condition are compared to other studies.

### Antimicrobials

Antimicrobial preparations are most frequently administered by the oral route in dogs (8, 10, 11) and our survey confirmed this finding. Most of the authors studying antimicrobials in dogs (7–9, 11, 12) reached the conclusion that amoxicillin with clavulanic acid was by far the most frequently used systemic antimicrobial and our survey showed the same result. Nevertheless, there are few clinical reasons that support such extensive use. The Danish antibiotic use guidelines for companion animal practice (13) only classified amoxicillin with clavulanic acid as a first option antibiotic for a short list of bacterial infections (pneumonia, furunculosis, otitis media, pyelonephritis, acute metritis, orchitis/epididymitis, and dacryocystitis) most of them infrequent in dogs. Guardabassi et al. (14) also compiled a similar list including pneumonia, central nervous system infections, pyelonephritis, and pyoderma produced by isolates of *Staphylococcus pseudintermedius* susceptible to amoxicillin with clavulanic acid, which is the only condition where amoxicillin with clavulanic acid is the first option in the Swedish guidelines (15) for the clinical use of antibiotics in the treatment of dogs and cats.

As in our survey, amoxicillin and cephalexin were among the most common systemically used antimicrobials after amoxicillin with clavulanic acid (7–11). Cephalexin was reported to be the most commonly prescribed drug for pyodermas (10, 16), traumatic wounds and surgical procedures (10). Consequently, the beta-lactams class (penicillins and cephalosporins) were at the top of the prescription list, both in the overall rank of systemic antimicrobials and for several specific conditions affecting the skin (10, 11, 17), gastrointestinal tract, eyes, respiratory system, musculoskeletal system (10, 11), genitourinary, and respiratory systems (17).

Ranked from most to least commonly prescribed, the antimicrobial classes following beta-lactams for systemic use differ between countries and show different patterns. In the UK (7, 8), the next most common were nitroimidazoles, lincosamides and macrolides, and fluoroquinolones. In the Nordic countries [Sweden and Norway (18); Finland (10)], amoxicillin with clavulanic acid was followed by trimethoprim-sulphonamides, macrolides and lincosamides, fluoroquinolones, and metronidazole. In an Italian study with dogs and cats (11), fluoroquinolones ranked second after beta-lactams. Lastly, in Australia (12), the most commonly used antimicrobials following amoxicillin with clavulanic acid were trimethoprim-sulphonamides, metronidazole, and fluoroquinolones. Our data in Madrid ranked fluoroquinolones and imidazole derivatives after beta-lactams.

Metronidazole was the most systemically used antimicrobial for enteritis/gastroenteritis in our study, in agreement with the European data of De Briyne and others (2014) (17), but not in Finland (10) or Italy (11). Digestive disorders were the most frequently recorded condition for metronidazole in all these studies, which is in agreement with the guidelines mentioned above (13, 15). According to the Swedish (15) and Danish (13) guidelines, there are few indications for antibiotic treatment of gastrointestinal diseases (such as acute haemorrhagic diarrhea, small intestinal bacterial overgrowth or antibiotic-responsive diarrhea), suggesting that most of these antimicrobial treatments should have been avoided in our surveyed sample.

Aminoglycosides, especially neomycin and tobramycin, were the most commonly used topical antimicrobial class in our study, mainly for treating eye and cutaneous conditions. However, the antimicrobials recommended as the first option for conjunctivitis are fusidic acid (13, 15), polymyxin, and oxytetracycline (for gramnegative rods) and erythromycin (for streptococci) (14).

Another common antimicrobial topical treatment is for ear infections, although the Swedish guidelines (15) recommend that “antibiotics should not be used to treat otitis conditions that are not actually infected with bacteria.” A similar approach is followed in the Danish guidelines (13) that only recommend antimicrobial therapy for bacterial-caused otitis externa and otitis media. In our survey, topical polymyxin B was the most widely used, followed by fluoroquinolones (topical and oral formulations), topical florfenicol and oral beta- lactams. Fluoroquinolones were the systemic antimicrobial most frequently used for ear infection in dogs and cats in Italy (11).

Although in our survey fluoroquinolones were not the most frequently used antimicrobial for any condition, the overall data ranked fluoroquinolones as the second most frequently used antimicrobial class. In Europe (17), fluoroquinolones ranked second for skin and genitourinary infections and third for respiratory diseases in dogs, but the situation certainly varies among countries. In Italy (11), data from dogs and cats together, fluoroquinolones were ranked second after beta-lactams (penicillins and cephalosporins). In the UK (8), the use of fluoroquinolones was lower than the use of beta-lactams, nitroimidazoles, and lincosamides. In Finland (10), use of fluoroquinolones was less than beta-lactams, trimethoprim-sulphonamides and macrolide and lincosamides.

These data highlight that certain antimicrobial classes are preferred in certain countries (17), which might be related to interlinked factors such as differences in the prevalence of diseases, antimicrobial resistance levels, existing guidelines on antibiotic prescription, authorized VMP or prescribing behavior.

Amoxicillin with clavulanic acid and fluoroquinolones are good examples of broad-spectrum antibiotics. Some authors (7, 12) believe that their high use suggests a low standard of diagnosis by the clinician. The infrequent use of bacterial culture and antimicrobial susceptibility testing found in our study has also been previously emphasized (3, 11, 19) and could be one of the reasons for the high use of broad-spectrum antibiotics for empiric treatments [Escher et al. (11)]. According to different authors (2, 3, 19), antimicrobial therapy based on antimicrobial susceptibility testing is mainly reserved for complicated cases or after a preliminary poor response. Equally, cytology [the “microscopic examination of smears of exudates or aspirates from the infected site”(12)] is another easy and valuable diagnostic tool for bacterial infection (12, 13, 15), rarely used according to our survey.

De Briyne et al. (3) analyzed information sources guiding antibiotic prescription across Europe showing that companion animal practitioners, apart from the Swedish, as well as colleagues within the food production sector, do not consider guidelines as among the most important sources. Indeed, among EU countries we only found guidelines in the English language for antibiotic prescription in companion animals from Sweden (15) and Denmark (13), as mentioned before. Guidelines from other countries, such as Australia (20), are also available. In addition, there are also specific guidelines [respiratory tract infections (21), urinary tract diseases (22), and superficial bacterial folliculitis (23)] of the Working Group of the International Society for Companion Animal Infectious Diseases, a chart with recommendations of the Federation of European Companion Animal Veterinary Associations (24) and those of Guardabassi and others (2008) in a book (14).

Differences between countries in the prevalence of the main bacterial infections in dogs (where antimicrobials are the first therapeutic option) are not documented but do not appear to be a major factor contributing to the dissimilarities in antimicrobial prescriptions between countries.

Surprisingly for us, we found few reports concerning the most common conditions treated with antimicrobials in dogs. Nonetheless, the uncertainties that we observed when studying the information on medical records helped us to appreciate the difficulties in coming to a proper diagnosis. Manual checking of clinical databases (7) confirmed the difficulties in obtaining a final diagnosis by veterinary surgeons and the need for a standardized nomenclature for recording clinical diagnoses.

Perioperative antimicrobial prescription, before, during or after surgical procedures (15) is also a controversial subject. An article from the USA (25) focuses the subject on the decreasing incidence of surgical site infection by the implementation of appropriate antimicrobial therapy. Whereas the Swedish guidelines (15) are highly restrictive and only recommend prophylactic antimicrobial prescription in the cases of dirty wounds, contaminated wounds “if the risk of infection is deemed to be considerable,” clean-contaminated wounds “if the operation is estimated to last more than one and a half to two hours” and in a short list of operations. The Danish guidelines (13) emphasize the dog's status and expected surgery as the main criteria and recommend that only high-risk patients should receive antibiotics [those having serious or life-threatening systemic diseases and those who are not expected to survive 24 hours without surgery (13)]. Rantala et al. (10) found that 12% of the prescriptions in their study were for postoperative treatment, while in our survey the figure was quite similar (9.4%), although most uses did not fulfill the Swedish guidelines (15).

Our results revealed a noticeable off-label use of VMP in dogs, mainly related to failure to comply with the SPC on dosage and indication of use. In addition, we detected the use of human products in 37.2% of cases that probably would not be entirely supported by the cascade procedure. Most of the conditions described have a veterinary product authorized for dogs in Spain. According to an EMA reflection paper (5), the proportion of use of human products in cats and dogs ranges from 13 to 80%, but it is not clear if the same procedure for assessing off-label use was applied in all surveys. For instance, Escher et al. (11) reported off-label use with regard to the species' indication (dogs or cats) in 23.8% of cases, most of them because of labeling of the product for human use. Compliance with the dosage recommended by the manufacturer (±20%) was 53.4%. Our finding regarding higher overdosage than underdosage has also been previously reported (16). Nevertheless, this estimation may be markedly skewed because of the comparison only with SPC and not current guidelines.

A different issue arises when both the veterinary and human products have exactly the same active substance and comparable indications for use in animals and humans. According to Table 1, there are several drugs where practitioners could prescribe either veterinary or human products but choose the human product because of its lower price as mentioned by Escher et al. in Italy (11). Although these should be considered as examples of off-label use, in our opinion the risk of encouraging antimicrobial resistant bacteria does not change if the active substance is the same and the posology is correctly adapted for dogs.

Our results confirm that a selective pressure for antimicrobial resistant bacteria in dogs is operating in the city of Madrid, which could increase the risk for owners and workers of colonization or even infection with resistant bacteria from pets (2). Potential measures to mitigate this risk would be the improvement of the prescription controls for antimicrobials by veterinary practitioners, reducing empiric treatments and promoting better use of hygiene measures (hands washing) for owners after every contact with animals.

In conclusion, although surveys in other Spanish cities are needed to confirm our findings, the pattern of antimicrobial prescription in dogs in our study is similar to that described in several European countries, and encompass the same two highly interconnected key features: a very high level of use of amoxicillin with clavulanic acid and a very low level of antibiotic sensitivity testing. Consequently, attempts should be made to improve both features at the same time. The feasibility of antibiotic sensitivity testing depends on the promptness of results and price, as well as on the promotion of its usefulness for everyday practice. Increased use of antibiotic sensitivity testing could potentially reduce the empiric prescription of broad-spectrum antimicrobials, such as amoxicillin with clavulanic acid or fluoroquinolones, in favor of other equally effective antimicrobials but less risky for public health.

## Author Contributions

BG-P performed sampling and data analysis and revised the drafted manuscript. MM designed the study, revised the analysis of data, and drafted manuscript.

### Conflict of Interest Statement

The authors declare that the research was conducted in the absence of any commercial or financial relationships that could be construed as a potential conflict of interest.
