# Supplementary material for: Antimicrobial Prescriptions for Dogs in the Capital of Spain
Source: Front Vet Sci. 2018 Dec 4;5:309. doi: 10.3389/fvets.2018.00309 (PMC6288439; doi:10.3389/fvets.2018.00309)
Supplement: Supplementary file 1 [file Data_Sheet_1.PDF]

UNIVERSIDAD COMPLUTENSE DE MADRID

FACULTAD DE VETERINARIA

DEPARTAMENTO DE SANIDAD ANIMAL

Código postal

Fecha Recogida:

Datos del animal:

Nombre: **Raza:** **Fecha consulta:**

Fecha de nacimiento: **Peso:** **Macho** **Hembra**

Diagnóstico:

Contexto de atención:

Quirófano **Pre quirófano** **Post quirófano** **Consulta**

Sintomatología:

Respiratorio **Piel** **Digestivo** **Oído** **Urinario** **Otros:**_

Cultivo *(SI/NO)* **Antibiograma** *(SI/NO)* **Fiebre** *(SI/NO)*

Otras pruebas:

DEPARTAMENTO DE SANIDAD ANIMAL
Avda. Puerta de Hierro, s/n. 28040 – MADRID
Telf. 91.394.37.05; Fax. 39.08 / E-mail:mamoreno@ucm.es

Tratamiento 1

Naturaleza del tratamiento:

Nuevo tratamiento **Cambio de tratamiento** **Receta oficial** **Otra** **En clínica**

Antibiótico:

Sistémico **Tópico** **Principio activo:** **Nombre comercial:** **Personas** **Animales**

Vía de administración: **Posología:** cada durante

Presentación: **Cantidad:** *(mg/kg)*

Otros

Tratamiento 2

Naturaleza del tratamiento:

Nuevo tratamiento **Cambio de tratamiento** **Receta oficial** **Otra** **En clínica**

Antibiótico:

Sistémico **Tópico** **Principio activo:** **Nombre comercial** **Personas** **Animales**

Vía de administración: **Posología:** cada durante

Presentación: **Cantidad:** *(mg/kg)*

Otros

DEPARTAMENTO DE SANIDAD ANIMAL
Avda. Puerta de Hierro, s/n. 28040 – MADRID
Telf. 91.394.37.05; Fax. 39.08 / E-mail:mamoreno@ucm.es
